# Corporate internal control, financial mismatch mitigation and innovation performance

**DOI:** 10.1371/journal.pone.0278633

**Published:** 2022-12-27

**Authors:** Xiao Li, Zhiquan Zhao

**Affiliations:** 1 Systems and Industrial Engineering Technology Research Center, Zhongyuan University of Technology, Zhengzhou, Henan, China; 2 School of Economics and Management, Zhongyuan University of Technology, Zhengzhou, Henan, China; University of Almeria, SPAIN

## Abstract

Based on the resource allocation optimization theory, from the perspective of internal control (IC) and financial mismatch jointly affecting technological innovation, this study selects the listed enterprises in China’s capital market from 2012 to 2020 as the sample, and explores the mechanism among IC, financial mismatch and technological innovation. The results show that effective IC significantly promotes corporate innovation, and mitigates financial mismatch. The mitigation of financial mismatch presents a significant mediating effect between effective IC and innovation output. In Discussion, this study finds that the effects are significantly reflected in non-state-owned enterprises, but not in state-owned enterprises. Finally, it is suggested to improve IC effectiveness continuously, to stimulate innovation vitality, optimize financial resources allocation, and foster new momentum for economic development. And it is suggested to facilitate the transmission effect that effective IC mitigates financial mismatch, and enhances innovation output. Also, the innovation activities in state-owned and non-state-owned enterprises should be coordinated to promote the steady and healthy development of the economy.

## 1. Introduction

Technological innovation is the primary driving force for development, and an important strategic underpinning for building a modern economic system. Only the innovation-driven economy can achieve sustained and high-quality development [1]. The innovation-driven development has become the core strategy of current economic operation. In transition economies, innovation presents a positive impact on corporate performance [2], and is a key driver for economic growth [3]. As an indicator to measure corporate core competitiveness, innovation performance represents corporate innovation level and internal potential. However, an investigation shows that enterprises’ R&D intensity is less than 1% at present in China, lower than 2.5% ~ 4.1% in developed countries [4]. Therefore, it is an important issue of modern corporate governance to explore the important factors affecting innovation performance, to reduce the resistance in innovation process, and promote innovation process essentially.

As an important internal governance mechanism, IC may promote innovation through reasonable risk control (i.e. the “reasonable control” hypothesis), or inhibit innovation due to excessive risk avoidance (i.e. the “hypercorrection” hypothesis). For IC and innovation, most scholars believe that enterprises with higher-quality IC have better innovation performance [5]. However, the increasing responsibilities of the board of directors and the management for IC effectiveness makes IC system more strict, which goes against the start and survival of innovation projects, and the tedious and strict control mechanism may not be conducive to developing innovation projects [6]. In 2008 and 2010, China Ministry of Finance, Securities Regulatory Commission, Audit Office, Banking Regulatory Commission, Insurance Regulatory Commission jointly issued the “Basic Norms for Corporate Internal Control” and “Supporting Guidelines for Corporate Internal Control” respectively, which clearly point out that IC is implemented by the board of directors, the board of supervisors, the management and all employees, to achieve control objectives. Effective IC is an important guarantee to achieve strategic objectives, and innovation is a part of strategic objectives. Then, at this stage, how does corporate IC affect innovation performance?.

Innovation activities cannot be separated from the support of financial resources. In accordance with the resource allocation optimization theory, limited and scarce financial resources should be allocated among different enterprises according to the size of external financing needs, so that the enterprises with higher external financing needs can get more financial resources, while those with lower external financing needs get less capital. Otherwise, there will be a misallocation of financial resources among different enterprises. The mismatch between financial resources’ allocation structure and efficiency is called financial mismatch [7]. In China, the financial market has a differentiated financing environment based on the social structure, and the financial mismatch exists significantly in the financial market [8]. The financial mismatch results in the financial market failing to allocate capital resources and share risks effectively. Then, as the core of internal governance, has effective IC mitigated the financial mismatch borne by enterprises? Is there a specific transmission mechanism among IC, financial mismatch, and innovation output? The answer to the above questions is of great theoretical and practical significance for improving innovation power.

China’s economic development has entered a new era, and the development objective has shifted from high-speed growth to high-quality development [9]. The realization of high-quality economic development is inseparable from the optimal capital allocation and continuous technological innovation. Technological innovation plays a strong role in promoting high-quality economic development [10]. It is urgent to strengthen the main position of China’s enterprises in innovation. Enterprises should have a clear innovation strategy and a sound organizational system to accelerate technological innovation [11]. From the perspective of IC and financial mismatch jointly affecting technological innovation, this study tries to deepen the understanding of the internal logic of the impacts of IC and financial mismatch on innovation performance.

The contributions are as follows. Firstly, more literatures explore pathways to enhance innovation performance based on differentiated economic background [12,13]. However, the conclusions may not be suitable for transition economies. Based on the background of China’s transition economy, this study provides beneficial reference for enhancing the capability of financial services to entities from IC construction. Secondly, Li (2020) [14] investigated the mediating mechanism of corporate social responsibilities between effective IC and innovation output. And Oláh et al. (2021) [15] explored the mediating effect of innovation between inter-organizational trust and financial performance. However, few literatures incorporated IC, financial mismatch and innovation output into the same framework. This study explores the mitigation of financial mismatch as a transmission pathway between IC and innovation performance, which is helpful to understand the mechanism of IC affecting innovation output. Thirdly, Li (2020) [14] explained the transmission mechanism among IC, corporate social responsibilities and innovation performance from property attributes. Ma et al. (2022) [16] explored that environmental investment plays a mediating role between IC and green innovation from property attributes. However, few literatures explored the transmission mechanism among IC, financial mismatch and innovation performance from properties. This study differentiates state-owned and non-state-owned enterprises, and explores the transmission effect of financial mismatch mitigation for the impact of effective IC on innovation performance, providing decision-making reference for enterprises with different attributes to improve innovation output.

The remaining parts are organized as follows. Section 2 presents Literature review, theoretical analysis, and research hypothesis; Section 3 provides Data source, variable definition and model setting; Section 4 carries Empirical analysis; Section 5 conducts Alleviating endogeneity; Section 6 carries Robustness test; Section 7 provides Discussion. And section 8 draws conclusions and provides recommendations.

## 2. Literature review, theoretical analysis, and research hypothesis

### 2.1 Effective IC and innovation performance

Innovation activities have a long cycle and higher risk, so the contract, supervision and agency costs are higher, and innovation information is more likely to be underestimated [17]. The intensity of innovation activities depends on managers’ judgment on the risks and expected benefits from R&D activities [18]. Innovation activities are uncertain, and the benefits cannot be realized in the short term [19]. Rational executives or risk averters may give up innovation activities because of uncertain innovation benefits. For instance, in Ghana, innovation capacity takes a negative and insignificant impact on innovation efficiency [12]. Corporate innovation is influenced by organization empowerment, intellectual property protection, corporate governance, and so on. Inclusive leadership affects innovation output positively and significantly in Saudi manufacturing firms [13]. In fact, corporate governance is the institutional foundation of innovation activities, and plays a key role in benefit distribution and power allocation [20]. As an internal governance mechanism, effective IC establishes a scientific decision-making system and power balance measure, timely corrects and prevents senior executives’ irrational behaviors [21], and encourages them to carry out innovation activities with strategic significance. Moreover, effective IC means that enterprises can reduce innovation risks through the overall control such as organizational structure, decision-making, implementation and supervision mechanisms [22]. In addition, R&D activities can be controlled at the business level through the procedures such as target setting, item identification, risk assessment, control activities, and the methods such as authorization approval, incompatible job separation, to ensure innovation activities’ actual results.

The “Basic Norms for Corporate Internal Control” “Supporting Guidelines for Corporate Internal Control” clearly mention that IC should promote enterprises to achieve strategic objectives. Within China’s enterprises, IC is a comprehensive risk management system that promotes the realization of development strategy [23]. The implementation of effective IC should follow the principles of comprehensiveness, importance, checks and balances, adaptability, and cost-effectiveness, to improve the management level and risk prevention capability, and play an important role in the selection and execution of innovation strategy [24]. IC is a dynamic management activity that keeps developing and changing [25], which contains and restrains, protects and guides, supervises and evaluates operation process. Then, effective IC produces high-quality internal information, and feeds the information to senior executives in time, to reduce the high risks generated by innovation activities through risk assessment and risk prevention [6], thus increases internal contracts’ execution efficiency. IC willingness lowers managerial overconfidence [26], reduces the possibility for managers to give up innovation projects while enjoying a “peaceful life,” decreases the resistance in innovation process, and effectively accelerates innovation output. Therefore, effective IC promotes innovation activities’ healthy and orderly development, and positively enhances innovation performance.

Based on the above analyses, the following research hypothesis is proposed.

**Hypothesis 1.** Effective IC significantly improves corporate innovation performance.

### 2.2 Effective IC and financial mismatch

The effective and reasonable allocation of financial resources is the important content and premise of realizing sustainable economic development. The focus of financial resources allocation is to reduce the misallocation, realize higher allocation efficiency, and promote the normal and healthy operation of the society and economy. However, the imperfection of financial market leads to the failure of capital allocation in the principle of equal capital marginal output among innovation subjects. In China, the allocation of financial resources is characterized by low efficiency and imbalance [27]. In the market with imperfect information, financial institutions tend to raise interest rates in order to reduce loan risks [19], thus leading to financial mismatch. And financial mismatch causes that scarce financial resources cannot be effectively allocated. Only by reducing capital mismatch, and improving financial efficiency and capacity utilization, can the economic growth be promoted better and faster.

As an internal supervision mechanism, effective IC can link enterprises and the market adequately [28,29], improve the quality of accounting information and financial reports [30,31], alleviate information asymmetry between enterprises and creditors, and reduce the risk assessment of creditors such as banks. And effective IC motivates the management to improve operational efficiency, and takes a significant and positive impact on financial performance [32], which enhances enterprises’ credibility in banks and other financial institutions, thus helps enterprises establish low-costs debt contracts. Moreover, effective IC improves the communication environment and business activities. Then, effective IC is beneficial for enterprises to shape reasonable cash policies that generate value creation [33], ease financing constraints, enhance financing efficiency, guard against capital encroachment, alleviate risk premiums, and reduce financing costs [34]. As a result, the internal supervision and motivation derived from effective IC provide a strong guarantee for implementing management decisions, which is conducive to promoting financing activities smoothly, enhancing the matching degree between financial resources’ allocation structure and efficiency, and mitigating the financial mismatch borne by enterprises.

Based on the above analyses, the following research hypothesis is proposed.

**Hypothesis 2.** Effective IC can significantly mitigate corporate financial mismatch.

### 2.3 Effective IC, financial mismatch and innovation performance

Effective IC accelerates corporate information flow and improves information quality [35], to a certain extent, alleviate information asymmetry, which is convenient for stakeholders to exercise supervision over the management’s illegal and irregular behaviors, effectively prevent and restrain the internal “trench”“tunnel” and other capital encroachment, ensure the legality and compliance of funds use, improve the effectiveness of capital acquisition, and reduce financial mismatch. However, the mismatch of limited and scarce financial resources may not only lead to frequent financing difficulties for the enterprises with stronger technological innovation intention and higher innovation efficiency, but also cause the distortion of price signals in the capital market, and the disorder of risk dispersion and income evaluation in the financial system, which hinder enterprises’ technological innovation activities and the improvement of innovation capability.

In current highly uncertain environment, the value of innovation is increasingly critical [36]. Technological innovation is a long-term and high-risk activity. For enterprises in transition economies, efficient financial market is an important factor affecting their innovation [37]. The stable and efficient financial supply is the foundation for innovation activities. External financing is the main capital source for independent innovation activities [38]. However, because of financial markets’ extensive growth, enterprises that can create high returns often face financing difficulties [39]. When carry out innovative activities, enterprises will encounter financing constraints if they are faced with insufficient internal funds, and cannot get external financing, or the external financing costs are higher. Financial mismatch exacerbates financing constraints, and external financing is restricted [40], which produce inhibition effect [41], increase the possibility of falling into liquidity dilemma, resulting in insufficient input in R&D activities [42], and inhibiting corporate R&D activities and innovation output [43,44]. In contrast, effective IC accelerates information flow, reduces risk evaluation from creditors, and avoids insufficient input in R&D activities caused by high financing costs [5]. Effective IC can be a useful channel of value protection [45], reducing the risk in innovation activities [35], to boost innovation momentum, and ensure orderly R&D activities. Further, considering the hypothesis that effective IC significantly mitigates financial mismatch (Hypothesis 2 above), this study argues that effective IC takes a positive impact on corporate innovation by mitigating financial mismatch. And a transmission pathway that IC mitigates financial mismatch, then improves innovation performance is formed.

Based on the above analyses, the following research hypothesis is proposed.

**Hypothesis 3.** The mitigation of financial mismatch takes a significant mediating effect between effective IC and innovation performance.

## 3. Data source, variable definition, and model setting

### 3.1 Data source

In 2012, China Securities Regulatory Commission issued the revised “Guidelines for Industry Classification of Listed Companies”. To facilitate industry classification, the listed companies publicly traded in China’s capital market from 2012 to 2020 are selected as the sample. The data are from Chinese Research Data Services Platform and Wind Financial Terminal. The following principles are followed for filtering data. Firstly, in view of the particularity of accounting methods in finance and insurance enterprises, they are removed from the sample. Secondly, to ensure the integrity and reliability of data, the annual observations with missing values or outliers are excluded. Thirdly, the enterprises processed by ST, *ST during the observed periods are removed. Finally, the financial data of 1844 enterprises are obtained as effective observations. To weaken the influence of extreme values, bidirectional 1% quantile Wionsorize is applied to continuous variables.

### 3.2 Variable definition

Table 1 shows the name and description for each variable.

**Table 1:** Variable name and description.

Nature	Symbol	Name	Calculation method
Explained variable	LnPATENT	Innovation performance	The natural logarithm of the sum of the number of patent applications and 1
Explanatory variable	IC	IC effectiveness	Dibo · IC Index of Listed Companies
Mediating variable	FMM	Financial mismatch	The ratio of an enterprise’s capital costs to the average capital costs of the industry in which the enterprise belongs
Control Variable	R&D	R&D input intensity	The percentage of R&D input in operating revenue
	LEV	Asset-liability ratio	Total liabilities/total assets
	ROA	Return on total assets	Net margin/average total assets; the average total assets are the average of total assets at the beginning and end of the period; the same as below
	TAT	Total assets turnover	Current operating income/average total assets
	SGR	Sales growth rate	(Current sales revenue—previous sales revenue)/previous sales revenue
	BDS	Board structure	The proportion of independent directors on the board of directors
	SHJZ	Ownership concentration	Shareholding ratio of the largest shareholder/that of the second largest shareholder
	Age	Corporate age	The periods from the establishment of the company to the end of the observed year
	LnSALARY	Executive compensation	The natural logarithm of the total annual salaries of directors, supervisors, and executives
	LnASSET	Corporate scale	The natural logarithm of total assets at the end
	AUDIT	Audit opinion	Dummy variable, 1 for the standard unqualified opinion; otherwise, 0
	STATE	Property attribute	Dummy variable, 1 for state-owned enterprises; 0 for non-state-owned enterprises
	YEAR	Year	Annual effect
	IND	Industry	Industry effect; in accordance with the revised “Guidelines for Industry Classification of Listed Companies”, a total of 17 industry dummy variables are set up according to categories
	ε		Random disturbance term

#### 3.2.1 Explained variable

In line with Fang et al. (2014) [46], Acharya and Xu (2017) [47], and Li et al. (2022) [48], this study adopts patent output to measure corporate innovation performance. The more patents applied in one year, the better the innovation performance. In accordance with “The Rules for the Implementation of Patent Law” in China, the patents are classified as inventions, utility models and appearance designs. Usually, it takes a certain amount of time for patents from application to be granted. Therefore, this study evaluates innovation performance mainly based on patent applications, and adopts patents granted in Discussion.

#### 3.2.2 Explanatory variable

For IC effectiveness, in 2011, Shenzhen Dibo Risk Management Technology Co., Ltd released the “Dibo · IC Index of Listed Companies” in China. The index is designed based on the realization of IC five objectives, namely compliance, reporting, asset safety, operation, strategy, and comprehensively reflects listed companies’ IC level and risk control ability. IC is not a simple sum of individual elements, but as a system in operation. IC effectiveness depends on the efficiency and effect of overall operation. Therefore, in line with Li (2022) [49], this study evaluates IC effectiveness in accordance with the comprehensive index of “Dibo · IC Index of Listed Companies”.

#### 3.2.3 Mediating variable

For financial mismatch (FMM), the capital costs borne by an enterprise higher than the average of the industry is regarded as the financial mismatch borne by the enterprise [50,51]. The greater the capital costs borne by an individual enterprise are higher than the average in the industry, the higher the degree of financial mismatch it bears. The financial mismatch borne by an enterprise can be reflected by the deviation of its capital costs from the average of the industry [51]. Thereout, with reference to Zhen and Luo (2019) [41], this study adopts the ratio of an enterprise’s interest rate to the average interest rate of the industry, in which the enterprise belongs, to reflect the financial mismatch borne by the enterprise. The interest rate borne by the enterprise is the ratio of the interests payable to the liabilities minus accounts payable. Eq (1) presents the calculation for FMM. Where, A_1_ and A_2_ represent interests payable and accounts payable, respectively; L represents total liabilities; and I_IND is the average interest rate of the industry.


FMM=(A1/(L‐A2))I_IND (1)

**3.2.4 Control variable.** With reference to Luong et al. (2017) [52], Zhong (2018) [43], Li and Liu (2021) [53], the following variables—R&D input intensity, Asset-liability ratio, Return on total assets, Total assets turnover, Sales growth rate, Board structure, Ownership concentration, Corporate age, Executive compensation, Corporate scale, Audit opinion, and Property attribute are controlled, to investigate their possible effects on innovation performance and financial mismatch, respectively. Also, Annual and Industry effects are controlled in regression.

### 3.3 Model setting-up

With reference to Li (2020) [14], and Li (2022) [49], the following panel regression Models 1, 2 and 3 are constructed, to verify Hypotheses 1, 2 and 3 above. For the control variables in Models 1 to 3, to weaken the endogeneity caused by reverse causality, R&D, LEV, ROA, TAT, SGR, LnSALARY, LnASSET, and AUDIT are taken as first-order lags in regression. And in Models 1 and 3, this study adopts the natural logarithm of the sum of the number of patent applications and 1 to measure innovation performance [54]; in Models 1 to 3, for the explained variable (IC), its value is equal to the “Dibo · IC Index of Listed Companies” divided by 1000 to be standardized, to avoid the adverse impact of excessive dimensional differences between the explained and explanatory variables on regression results.

**Model 1**.


$$\begin{array}{l} LnPATENT_{i,t}=\alpha_{0}+\alpha_{1}IC_{i,t}+\alpha_{2}R\&D_{i,t-1}+\alpha_{3}LEV_{i,t-1}+\alpha_{4}ROA_{i,t-1}+\alpha_{5}TAT_{i,t-1}+\alpha_{6}SGR_{i,t-1}+\\ \;\alpha_{7}BDS_{i,t}+\alpha_{8}SHJZ_{i,t}+\alpha_{9}Age_{i,t}+\alpha_{10}LnSALARY_{i,t-1}+\alpha_{11}LnASSET_{i,t-1}+\\ \;\alpha_{12}AUDIT_{i,t-1}+\alpha_{13}STATE_{i,t}+\alpha_{14}\sum_{t}YEAR+\alpha_{15}\sum_{t}IND+\varepsilon_{i,t} \end{array}$$ (2)

**Model 2**.


$$\begin{array}{l} FMM_{i,t}=\beta_{0}+\beta_{1}IC_{i,t}+\beta_{2}R\&D_{i,t-1}+\beta_{3}LEV_{i,t-1}+\beta_{4}ROA_{i,t-1}+\beta_{5}TAT_{i,t-1}+\beta_{6}SGR_{i,t-1}+\beta_{7}BDS_{i,t}+\\ \;\beta_{8}SHJZ_{i,t}+\beta_{9}Age_{i,t}+\beta_{10}LnSALARY_{i,t-1}+\beta_{11}LnASSET_{i,t-1}+\beta_{12}AUDIT_{i,t-1}+\\ \;\beta_{13}STATE_{i,t}+\beta_{14}\sum_{t}YEAR+\beta_{15}\sum_{t}IND+\varepsilon_{i,t} \end{array}$$ (3)

**Model 3**.


$$\begin{array}{l} LnPATENT_{i,t}=\delta_{0}+\delta_{1}IC_{i,t}+\delta_{2}FMM_{i,t}+\delta_{3}R\&D_{i,t-1}+\delta_{4}LEV_{i,t-1}+\delta_{5}ROA_{i,t-1}+\delta_{6}TAT_{i,t-1}+\\ \;\delta_{7}SGR_{i,t-1}+\delta_{8}BDS_{i,t}+\delta_{9}SHJZ_{i,t}+\delta_{10}Age_{i,t}+\delta_{11}LnSALARY_{i,t-1}+\\ \;\delta_{12}LnASSET_{i,t-1}+\delta_{13}AUDIT_{i,t-1}+\delta_{14}STATE_{i,t}+\\ \;\delta_{15}\sum_{t}YEAR+\delta_{16}\sum_{t}IND+\varepsilon_{i,t} \end{array}$$ (4)

**Fig 1:**
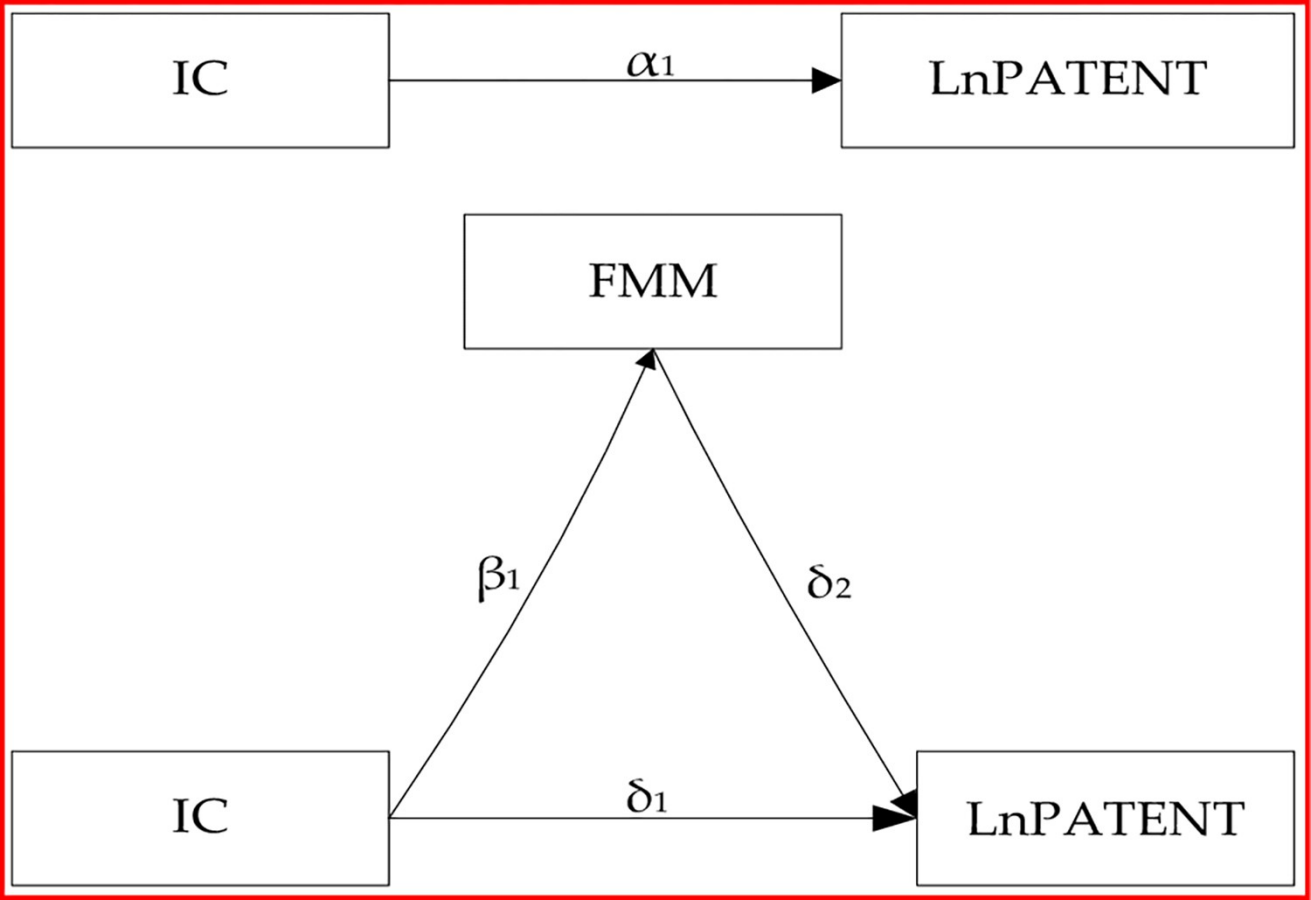
The mediation effect diagram.

In Model 1, the coefficient α_1_ represents the total effect of IC on innovation performance. In Model 2, the coefficient β_1_ is the effect of IC on financial mismatch. In Model 3, the coefficient δ_1_ represents the direct effect of IC on innovation performance, and δ_2_ represents the effect of financial mismatch on innovation performance. If δ_2_ is significantly negative, it indicates that the mitigation of financial mismatch promotes corporate innovation. If the absolute value of δ_1_ is less than that of α_1_, it indicates that the mitigation of financial mismatch plays a mediating role between effective IC and innovation performance. In other words, effective IC promotes corporate innovation by mitigating financial mismatch. In accordance with Wen and Ye (2014) [55], and the testing process of mediation effect can be described by the mediation effect diagram shown in Fig 1.

## 4. Empirical analysis

### 4.1 Descriptive statistics

Table 2 presents the descriptive statistics. For the explained variable, the maximum (minimum) of LnPATENT is 7.014 (0.000), and the median (mean) is 3.045 (2.948), indicating that there is a big gap in different enterprises’ innovation performance, and some enterprises’ innovation output needs to be improved. For the explanatory variable, the mean (standard deviation) of IC is 0.635 (0.137). Overall, IC effectiveness is better. However, IC effectiveness varies greatly among different enterprises. For the mediating variable, the maximum (minimum) of FMM is 3.945 (0.000), and the mean (median) is 1.028 (0.978). There is an obvious financial mismatch in enterprises, and the financial mismatch shows an obvious difference. Financial mismatch may reduce the sensitivity of the financial market to identify innovation projects, resulting in the misplacement effect of innovation resources, thereby exacerbating the risk of technological innovation [51].

**Table 2:** Descriptive statistics.

Variable	Mean	Median	Maximum	Minimum	Deviation	Observations
LnPATENT	2.948	3.045	7.014	0.000	1.624	15071
IC	0.635	0.664	0.825	0.000	0.137	15071
FMM	1.028	0.978	3.945	0.000	0.794	15071
R&D	4.307	3.410	25.180	0.020	4.334	15071
LEV	41.986	41.345	86.682	5.359	19.779	15071
ROA	3.800	3.508	21.065	-22.088	5.982	15071
TAT	0.654	0.562	2.554	0.108	0.418	15071
SGR	12.142	8.593	144.018	-48.921	28.813	15071
BDS	0.376	0.333	0.571	0.333	0.054	15071
SHJZ	8.865	3.616	89.665	1.004	14.179	15071
Age	19.284	19.003	35.584	8.085	5.401	15071
LnSALARY	15.311	15.274	17.253	13.629	0.717	15071
LnASSET	22.371	22.191	26.302	20.074	1.280	15071
AUDIT	0.977	1.000	1.000	0.000	0.150	15071
STATE	0.365	0.000	1.000	0.000	0.481	15071

For the control variables, the mean of R&D is 4.31%, and the maximum (minimum) is 25.18% (0.02%). R&D input accounts for a lower proportion in operating revenue. Moreover, there is a distinct gap in R&D input, and some enterprises are not enthusiastic about technological innovation. The maxima (minima) of LEV, ROA, TAT, LnSALARY, and LnASSET are 86.68% (5.36%), 21.07% (-22.09%), 2.554 (0.108), 17.253 (13.629), and 26.302 (20.074). There are significant differences in sample enterprises’ size, and the debt pressure, asset earnings, asset turnover and executive compensation show significant differences. The median of SGR is 8.59%, lower than its mean (12.14%), implying that many enterprises’ sales growth has not reached the average of the market, and their sale growth is worrying.

The minimum of BDS is 0.333, in line with the provision that “at least one third of the board members of listed companies should be independent directors,” issued by China Securities Regulatory Commission. The mean of SHJZ is 8.87, suggesting the “sole majority shareholder” exists in China’s listed companies. Besides, the sample enterprises’ average age is equal to 19.284, and state-owned enterprises accounts for 36.50% on average. For the legitimacy and fairness of annual financial reports, external auditors hold a positive attitude towards most enterprises, which ensures the reliability of the data used in this study. On the whole, the sample has a good discrimination, providing a beneficial basis for regression.

### 4.2 Correlations

Table 3 reports the pairwise correlations. In Models 1 and 3, IC is positively and significantly correlated with LnPATENT (0.131, *p*< 0.01), which initially supports Hypothesis 1, suggesting that innovation performance can be improved when IC is more effective. In Model 2, IC is negatively and significantly correlated with FMM (-0.157, *p*< 0.01), which initially supports Hypothesis 2, implying that effective IC is helpful to alleviate financial mismatch. In Model 3, FMM is negatively and significantly correlated with LnPATENT (-0.033, *p*< 0.01), suggesting that financial mismatch takes an obvious adverse effect on technological innovation.

**Table 3:** Pairwise correlations.

Variable	LnPATENT	IC	FMM	R&D	LEV	ROA	TAT	SGR	BDS	SHJZ	Age	LnSALARY	LnASSET	AUDIT	STATE
LnPATENT	1.000														
IC	0.131***	1.000													
FMM	-0.033***	-0.157***	1.000												
R&D	0.096***	0.001	-0.076***	1.000											
LEV	0.188***	-0.100***	0.270***	-0.276***	1.000										
ROA	0.065***	0.314***	-0.242***	-0.027***	-0.299***	1.000									
TAT	0.040***	0.124***	-0.015*	-0.234***	0.175***	0.124***	1.000								
SGR	0.017**	0.040***	-0.022***	0.015*	0.000	0.079***	0.025***	1.000							
BDS	0.013*	0.017**	0.007	0.054***	0.000	-0.019**	-0.035***	0.005	1.000						
SHJZ	-0.024***	-0.019**	-0.021**	-0.087***	0.109***	-0.036***	0.057***	-0.029***	0.012	1.000					
Age	0.021***	-0.078***	0.003	-0.079***	0.133***	-0.029***	0.026***	-0.005	-0.056***	-0.032***	1.000				
LnSALARY	0.265***	0.125***	-0.030***	0.035***	0.143***	0.143***	0.086***	0.001	-0.038***	-0.078***	0.197***	1.000			
LnASSET	0.409***	0.129***	0.042***	-0.222***	0.524***	0.037***	0.091***	0.004	0.035***	0.072***	0.176***	0.458***	1.000		
AUDIT	0.065***	0.335***	-0.094***	-0.030***	-0.111***	0.195***	0.040***	0.028***	0.014*	-0.001	-0.052***	0.041***	0.033***	1.000	
STATE	0.090***	-0.001	-0.049***	-0.190***	0.304***	-0.068***	0.087***	-0.046***	-0.025***	0.200***	0.133***	0.050***	0.357***	0.027***	1.000

Note

*** Significant at 1%

** Significant at 5%

* Significant at 10%.

For the control variables, in Models 1 and 3, R&D (0.096), LEV (0.188), ROA (0.065), TAT (0.040), Age (0.021), LnSALARY (0.265), LnASSET (0.409), AUDIT (0.065), and STATE (0.090) are positively and significantly correlated with LnPATENT (*p*< 0.01); as are SGR (0.017, *p*< 0.05), and BDS (0.013, *p*< 0.10). And SHJZ is negatively and significantly correlated with LnPATENT (-0.024, *p*< 0.01). In Model 2, LEV (0.270), LnASSET (0.042) are positively and significantly correlated with FMM (*p*< 0.01). And R&D (-0.076), ROA (-0.242), SGR (-0.022), LnSALARY (-0.030), AUDIT (-0.094), and STATE (-0.049) are negatively and significantly correlated with FMM (*p*< 0.01); as are SHJZ (-0.021, *p*< 0.05), and TAT (-0.015, *p*< 0.10). These correlations ensure the rationality of Models 1, 2 and 3 above. Besides, the maximum correlation is 0.524, existing between LEV and LnASSET, less than the threshold of 0.800. The serious multicollinearity does not exist in Models 1, 2 and 3, providing a reliable guarantee for regression.

### 4.3 Model regression analysis

Because other factors influencing the explained variables are not controlled, the descriptive statistics and correlations are only preliminary analysis results. The data type adopted in this study is Panel data. The regression analyses for Panel data mainly include the mixed OLS method, fixed effects model and random effects model. The fixed effects regression alleviates the interference caused by unobservable variables that do not change with time, and presents a certain information advantage.

LSDV method is adopted to test Models 1, 2 and 3, rejecting the hypothesis that the coefficients on all individual dummy variables are zero, indicating the existence of individual fixed effects. The robust Hausman tests for fixed and random effects show that Sargan-Hansenχ^2^ statistics are 240.376 (*p* = 0.000), 252.011 (*p* = 0.000), and 233.751 (*p* = 0.000) respectively, suggesting that the fixed effects model should be adopted. In Table 4, columns 1, 2 and 3 reports the regression results for Models 1, 2 and 3 in turn.

**Table 4:** Regression results for Models 1–3.

Variable	(1)	(2)	(3)
	Model 1	Model 2	Model 3
	Coef. (S.E.)	Coef. (S.E.)	Coef. (S.E.)
Intercept	12.707*** (0.992)	-2.564*** (0.680)	12.506*** (0.998)
IC	0.179** (0.080)	-0.303*** (0.056)	0.156** (0.079)
FMM			-0.078*** (0.021)
L.R&D	0.010 (0.006)	-0.003 (0.002)	0.009 (0.006)
L.LEV	-0.001 (0.001)	0.021*** (0.001)	0.000 (0.001)
L.ROA	0.010*** (0.002)	-0.004*** (0.001)	0.009*** (0.002)
L.TAT	0.003 (0.058)	-0.119*** (0.037)	-0.006 (0.058)
L.SGR	0.004 (0.005)	-0.000 (0.009)	0.004 (0.005)
BDS	-0.443 (0.283)	0.135 (0.166)	-0.432 (0.283)
SHJZ	-0.062 (0.053)	-0.043 (0.033)	-0.066 (0.052)
Age	-0.860*** (0.023)	0.016 (0.012)	-0.859*** (0.023)
L.LnSALARY	0.033 (0.021)	0.009 (0.015)	0.034 (0.021)
L.LnASSET	0.391*** (0.039)	0.095*** (0.028)	0.398*** (0.039)
L.AUDIT	0.154* (0.084)	-0.088 (0.060)	0.147* (0.084)
STATE	-0.055 (0.087)	-0.130** (0.058)	-0.065 (0.086)
YEAR/IND	YES	YES	YES
# of obs.	12966	12966	12966
Within_R^2^	0.193	0.184	0.194
F_Value	77.75***	73.62***	76.45***

Note

*** Significant at 1%

** Significant at 5%

* Significant at 10%.

Robust standard errors in brackets are clustered at corporate level.

#### 4.3.1 Analyses of Model 1’s regression results

In column 1, the coefficient on IC is positive and significant (0.179, *p*< 0.05), indicating that effective IC has a significant promoting effect on innovation performance, which is consistent with the research of Chan et al. (2021) [56]. And this result supports the “reasonable control” hypothesis that effective IC promotes corporate innovation. Hypothesis 1 above is verified. Effective IC can identify, assess and respond to the risks brought by innovation activities [57], which can promote enterprises to be more willing to carry out innovation projects with higher risks, thereby fully enhance the efficiency of innovation output.

For the control variables, the coefficient on L.ROA is positive and significant (0.010, *p*< 0.01), indicating that the enterprises with stronger asset profitability are more capable of carrying out innovation activities, and significantly enhance innovation output. The coefficient on L.LnASSET is positive and significant (0.391, *p*< 0.01). Large-scale enterprises have more capital, personnel and technical reserves for R&D business, forming a certain scale effect, and their innovation ability and success probability are higher, so the efficiency of innovation output is higher. Also, the coefficient on L.AUDIT is positive and significant (0.154, *p*< 0.10). A positive audit evaluation motivates enterprises to carry out R&D activities to improve innovation efficiency. However, the coefficient on Age is negative and significant (-0.860, *p*< 0.01). From the perspective of life cycle, when a company has been in continuous operation for a long time and enters recession stage, its innovation activities are difficult to achieve innovation results, thus hindering innovation efficiency. Enterprises have differentiated priorities at different development stages, so they should carry out innovation activities at appropriate times to promote own sustainable and healthy development. The coefficients on the remaining control variables are not statistically significant.

#### 4.3.2 Analyses of Model 2’s regression results

In column 2, the coefficient on IC is negative and significant (-0.303, *p*< 0.01), indicating that effective IC can alleviate corporate financial mismatch. Hypothesis 2 above is verified. Effective IC reduces the information asymmetry between lenders and borrowers, corrects the distorted lending system, enables enterprises to obtain debt funds at lower costs, eases the “financing difficulties” and “high financing costs”, and then effectively mitigates the degree of financial mismatch.

For the control variables, the coefficients on L.ROA, L.TAT, and STATE are negative and significant (-0.004, *p*< 0.01; -0.119, *p*< 0.01; -0.130, *p*< 0.05). Good asset turnover and accounting earnings are conducive to easing financial mismatch. And compared with non-state-owned enterprises, state-owned enterprises face a higher degree of administrative intervention, enjoy more preferential interest rates, and often have a lower financial mismatch. However, the coefficients on L.LEV, and L.LnASSET are positive and significant (0.021, *p*< 0.01; 0.095, *p*< 0.01). The debt burden aggravates financing constraints, thus increasing credit financing costs and aggravating financial mismatch. And large-scale enterprises’ internal business processes are complicated, and there is a higher information asymmetry between them and creditors. Creditors may reduce loan amounts or increase risk return rates, to reduce own capital security risks, thus leading to a distinct increase in financial mismatch. The coefficients on the remaining control variables are not statistically significant.

#### 4.3.3 Analyses of Model 3’s regression results

In column 3, the coefficient on IC is positive and significant (0.156, *p*< 0.05). Enterprises improve IC effectiveness, so that they can resist risks brought by innovation uncertainty, and then enhance innovation output. The coefficient on FMM is negative and significant (-0.078, *p*< 0.01). Financial mismatch takes a significant and negative effect on innovation performance. Financial mismatch not only affects enterprises’ resource allocation and behavioral choice preference, but also directly reduces the efficiency of using financial resources. The higher the degree of financial mismatch is, the lower the level of patent output is, which is not conducive to improving innovation efficiency. Meanwhile, considering that the coefficients on IC in Models 1 and 2 are significant (*p*< 0.05; *p*< 0.01), this study holds that the mitigation of financial mismatch has a significant mediating effect between effective IC and innovation performance. Effective IC has always been regarded as an important measure to improve the management level and crisis response ability. Enterprises reinforce IC construction to standardize control process, reduce information asymmetry, principal-agent and other problems. Thereby, effective IC reduces debt financing costs, promotes resources optimal allocation, mitigates financial mismatch, and enhances innovation performance. Hypothesis 3 above is verified. With reference to Wen and Ye (2014) [55], the non-parametric percentile bootstrap (1000) method for deviation correction is adopted. Further, the confidence interval of β_1_×δ_2_ with 95% confidence is estimated to be [0.037, 0.082], where β_1_×δ_2_ is the product of the effects of IC on FMM, and FMM on LnPATENT. And the mediating effect size is approximately 13.20% (β_1_×δ_2_/α_1_ = (-0.303)×(-0.078)/0.179). The mediating effect test supports the positive effect of effective IC on corporate innovation by mitigating financial mismatch.

For the control variables, the coefficients on L.ROA, Age, L.LnASSET, and L.AUDIT are statistically significant (*p*< 0.01; *p*< 0.10), and the conclusions on them are consistent with those from Model 1.

## 5. Alleviating endogeneity

### 5.1 Tests based on Heckman two-step model

As an internal governance mechanism, effective IC is an important guarantee to achieve strategic objectives. However, the governance and the management may selectively invest resources in IC construction based on corporate fundamentals. The management may override IC, and thus leading to IC failure. Therefore, enterprises have a certain degree of “self-selection” in IC construction. To alleviate the endogeneity caused by the “self-selection” problem, Heckman (1979) two-step model is adopted to conduct tests.

In the first step, the Probit Model 4 on IC effectiveness is constructed, and the inverse Mills ratio (IMR) is estimated. In line with Li (2020) [14], this study sets a dummy variable for IC effectiveness, which is expressed as DIC. If corporate DIB · IC index in the current year is higher than that in the previous year, it indicates that IC effectiveness tends to improve, and DIC is 1; otherwise, DIC is 0, indicating that IC effectiveness remains unchanged or decreases. Meanwhile, with reference to Doyle et al. (2007) [58], Zhang et al. (2016) [59], Wang and Dai (2019) [5], this study adopts whether the company is audited by the Big Four accounting firms (Big4, 1 for yes; 0 for no), and whether the company is dealt with in violation of regulations by China Ministry of Finance, Securities Regulatory Commission and Stock Exchanges (Isviolated, 1 for yes; 0 for no) as the instrumental variables. Usually, the audit conducted by the Big Four accounting firms forms a supervision effect on IC construction. And if the company is dealt with in violation of regulations, it means that IC effectiveness is relatively lower. Therefore, Big4, Isviolated are highly related with DIC. But the audit conducted by the Big Four accounting firms, and the penalties for violations will not directly affect corporate innovation performance and financial mismatch. Then, the first-order lags of Big4 and Isviolated (i.e. L.Big4 and L.Isviolated) are introduced into Model 4, to meet the exclusion restriction for Heckman model.

**Model 4**.


$$\begin{array}{l} DIC_{i,t}=\zeta_{0}+\zeta_{1}R\&D_{i,t-1}+\zeta_{2}LEV_{i,t-1}+\zeta_{3}ROA_{i,t-1}+\zeta_{4}TAT_{i,t-1}+\zeta_{5}SGR_{i,t-1}+\zeta_{6}BDS_{i,t}+\zeta_{7}SHJZ_{i,t}+\\ \;\zeta_{8}Age_{i,t}+\zeta_{9}LnSALARY_{i,t-1}+\zeta_{10}LnASSET_{i,t-1}+\zeta_{11}AUDIT_{i,t-1}+\zeta_{12}STATE_{i,t}+\\ \;\zeta_{13}Big4_{i,t-1}+\zeta_{14}Isviolated_{i,t-1}+\zeta_{15}\sum_{t}YEAR+\zeta_{16}\sum_{t}IND+\varepsilon_{i,t} \end{array}$$ (5)

In the second step, the IMR estimated in the first step is added into the original Models 1, 2 to 3 as the control variable, respectively. Moreover, compared with utility models and appearance designs, invention patents are more innovative, and may better represent the actual technological development [60]. Further, the application number of invention patents is added by 1, and the natural logarithm is taken and expressed as LnINVENT, to represent innovation output efficiency. On this basis, the impacts of IC on innovation efficiency and financial mismatch are examined again.

As shown in Table 5, in the first step, the coefficients on L.Big4, and L.Isviolated are statistically significant (*p*< 0.10 or *p*< 0.01), indicating that the variables introduced into the selection regression are valid. In the second step, from columns 1 to 3, the coefficients on IMR are statistically significant (*p*< 0.01 or *p*< 0.05), indicating that there is a certain degree of “self-selection” in the observations. The Heckman two-step model can alleviate the regression bias caused by the “self-selection” problem.

**Table 5:** Test results for heckman two-step model.

Variable	(1)	(2)	(3)
	Model 1	Model 2	Model 3
	Coef. (S.E.)	Coef. (S.E.)	Coef. (S.E.)
Intercept	-14.420*** (0.602)	1.458*** (0.303)	-14.239*** (0.597)
IC	0.050** (0.023)	-0.080*** (0.012)	0.040* (0.023)
FMM			-0.124*** (0.024)
L.R&D	0.067*** (0.004)	-0.008*** (0.002)	0.066*** (0.004)
L.LEV	0.000 (0.001)	0.018*** (0.001)	0.003* (0.001)
L.ROA	-0.002 (0.006)	-0.008*** (0.003)	-0.003 (0.006)
L.TAT	0.111*** (0.038)	-0.149*** (0.019)	0.092** (0.038)
L.SGR	0.008 (0.016)	0.037*** (0.008)	0.013 (0.016)
BDS	-0.239 (0.317)	0.323** (0.160)	-0.199 (0.314)
SHJZ	-0.002** (0.001)	-0.001*** (0.000)	-0.002** (0.001)
Age	-0.004 (0.004)	-0.004** (0.002)	-0.004 (0.004)
L.LnSALARY	0.125*** (0.025)	0.014 (0.012)	0.127*** (0.024)
L.LnASSET	0.561*** (0.021)	-0.020* (0.010)	0.558*** (0.020)
L.AUDIT	0.372*** (0.127)	0.082 (0.064)	0.383*** (0.125)
STATE	0.140*** (0.043)	-0.262*** (0.021)	0.107** (0.043)
IMR	1.054*** (0.331)	-0.338** (0.167)	1.012*** (0.328)
YEAR/IND	YES	YES	YES
# of obs.	12933	12933	12933
L.Big4	0.100* (0.053)	0.100* (0.053)	0.100* (0.053)
L.Isviolated	0.230*** (0.034)	0.230*** (0.034)	0.230*** (0.034)
Wald_chi^2^	2593.61***	1681.70***	2669.24***

Note

*** Significant at 1%

** Significant at 5%

* Significant at 10%.

In the second step, from Model 1 to 3, the coefficients on IC are statistically significant (0.050, *p*< 0.05; -0.080, *p*< 0.01; 0.040, *p*< 0.10). Again, these results indicate that effective IC enhances innovation output and alleviates financial mismatch significantly. Hypotheses 1 and 2 are verified again. In Model 3, the coefficient on FMM is negative and significant (-0.124, *p*< 0.01). Financial mismatch leads to the failure of resources optimal allocation, which hinders innovative activities, and takes an adverse effect on invention patents with higher-quality innovation. Based on the coefficients on IC from Models 1 to 3, and the coefficient on FMM in Model 3, this study believes that effective IC eases the information asymmetry between creditors and enterprises, reduces the threshold and costs of financial services, to serve the innovation projects accurately, and enhance the output efficiency of technological innovation. Thereout, the mitigation of financial mismatch presents a significant mediating effect between effective IC and innovation performance. Again, Hypothesis 3 is verified.

For the control variables, in Models 1 and 3, the conclusions on L.LnASSET, and L.AUDIT are consistent with those from Table 4. Besides, the coefficients on L.R&D are positive and significant (0.067, *p*< 0.01; 0.066, *p*< 0.01); as are those on L.TAT (0.111, *p*< 0.01; 0.092, *p*< 0.05), and L.LnSALARY (0.125, *p*< 0.01; 0.127, *p*< 0.01). The enhancement of R&D input and good asset turnover promotes innovation activities. The higher compensation is a recognition for senior executives’ work, and enhances their confidence and motivation to facilitate innovation [61]. Also, the coefficients on STATE are positive and significant (0.140, *p*< 0.01; 0.107, *p*< 0.05). State-owned enterprises tend to be larger. As shown in Table 3, STATE is positively and significantly correlated with LnASSET (0.357, *p*< 0.01). In larger enterprises, the capital and personnel advantages increase innovation performance. However, the coefficients on SHJZ are negative and significant (-0.002, *p*< 0.05; -0.002, *p*< 0.05). Probably, the “sole majority shareholder” leads to a serious benefit encroachment, which negatively affects innovation output. In Model 3, the coefficient on L.LEV is positive and significant (0.003, *p*< 0.10), suggesting that creditor governance effect enhances innovation performance.

In Model 2, the conclusions on L.LEV, L.ROA, L.TAT, and STATE are consistent with those from Table 4. However, the coefficient on L.LnASSET is negative and significant (-0.020, *p*< 0.10), which differs that from Table 4. Perhaps, larger enterprises’ scale effect enhances financing advantage, thus weaken financial mismatch to a certain extent. Besides, the coefficients on L.R&D, and SHJZ are negative and significant (-0.008, *p*< 0.01; -0.001, *p*< 0.01). Within enterprises, the higher accumulation of R&D input reduces the management and coordination costs, and alleviates the risks in R&D process [62], thus mitigating financial mismatch. And perhaps, due to higher shareholding ratio, the largest shareholder’s individual interests tend to be consistent with corporate overall interests, and its governance effect alleviates financial mismatch. Also, the coefficient on Age is negative and significant (-0.004, *p*< 0.05), suggesting that the enterprises in growth stage can quickly respond to the changes in external environment to mitigate financial mismatch. However, the coefficient on L.SGR is positive and significant (0.037, *p*< 0.01), suggesting that the rapid and irrational growth aggravates operation risks, and increases debt financing costs, leading to financial mismatch. And the coefficients on BDS is positive and significant (0.323, *p*< 0.05), implying that it is necessary to enhance independent directors’ supervision efficiency, to further mitigate financial mismatch.

### 5.2 Instrumental variable method

There may be reverse causality between innovation performance and IC effectiveness, financial mismatch and IC effectiveness, which may lead to endogeneity. The enterprises with higher innovation output and lower financial mismatch have better fundamentals, adequate resources to invest in IC construction, and improve IC effectiveness. To weaken the interference from the endogeneity, the instrumental variable method is adopted. Appropriate instrumental variables are highly correlated with the endogenous variable, and not with explained variables. With reference to Doyle et al. (2007) [58], Zhang et al. (2016) [59], Wang and Dai (2019) [5], Big4 and Isviolated are introduced as the instrumental variables. Big4 and Isviolated have the same meanings as in Section 5.1. Further, this study carries out Two Stage Least Square (2SLS) regression. In the first stage, the first-order lags of Big4 and Isviolated are controlled, to examine the relations between IC and Big4, IC and Isviolated. Table 6 reports the test results for 2SLS regression.

**Table 6:** Test results for instrumental variable method.

Variable	(1)	(2)	(3)
	Model 1	Model 2	Model 3
	Coef. (S.E.)	Coef. (S.E.)	Coef. (S.E.)
Intercept	-13.345*** (0.393)	1.423*** (0.225)	-13.200*** (0.392)
IC	0.251** (0.118)	-0.382*** (0.072)	0.219* (0.123)
FMM			-0.102*** (0.025)
L.R&D	0.054*** (0.007)	-0.004* (0.002)	0.053*** (0.007)
L.LEV	0.006*** (0.001)	0.014*** (0.001)	0.007*** (0.001)
L.ROA	0.009** (0.004)	-0.006** (0.002)	0.008** (0.004)
L.TAT	0.036 (0.046)	-0.046* (0.026)	0.029 (0.045)
L.SGR	0.022* (0.013)	0.017 (0.020)	0.024* (0.012)
BDS	-0.455** (0.216)	0.329*** (0.127)	-0.425* (0.217)
SHJZ	-0.003*** (0.001)	-0.002*** (0.000)	-0.003*** (0.001)
Age	-0.005* (0.002)	-0.004*** (0.001)	-0.005** (0.002)
L.LnSALARY	0.122*** (0.026)	0.026** (0.013)	0.124*** (0.025)
L.LnASSET	0.523*** (0.023)	0.022 (0.014)	0.524*** (0.023)
L.AUDIT	0.082 (0.281)	0.772*** (0.176)	0.144 (0.291)
STATE	0.038 (0.029)	-0.277***(0.017)	0.010 (0.029)
YEAR/IND	YES	YES	YES
# of obs.	12933	12933	12933
Wald_chi^2^	6186.41***	1954.26***	6284.90***
L.Big4	0.241*** (0.053)	0.241*** (0.053)	0.221*** (0.053)
L.Isviolated	-0.275*** (0.039)	-0.275***(0.039)	-0.264*** (0.039)
F_Value	36.530***	36.530***	32.910***

Note

*** Significant at 1%

** Significant at 5%

* Significant at 10%.

Robust standard errors in brackets are clustered at corporate level.

In Table 6, the coefficients on L.Big4, and L.Isviolated are the first-stage results, which are significant statistically (*p*< 0.01). In the last row, the F-values for the weak instrumental variable tests are far greater than 10.000, implying that there are no weak instrumental variables [63]. In the second-stage regression, from Models 1 to 3, the coefficients on IC are significant (0.251, *p*< 0.05; -0.382, *p*< 0.01; 0.219, *p*< 0.10). In Model 3, the coefficient on FMM is negative and significant (-0.102, *p*< 0.01). Again, these results indicate that effective IC promotes innovation output, and reduces financial mismatch. Moreover, financial mismatch presents an inhibition effect on innovation performance. Effective IC alleviates financial mismatch, and then improves innovation performance. The mitigation of financial mismatch takes a significant mediating effect between IC and innovation performance. The size of this mediating effect is estimated to be about 15.52% (β_1_×δ_2_/α_1_ = (-0.382)×(-0.102)/0.251). Hypotheses 1, 2, and 3 are verified again.

For the control variables, in Models 1 and 3, the conclusions on L.R&D, L.LEV, L.ROA, SHJZ, Age, L.LnSALARY, and L.LnASSET are consistent with those from Tables 4 or 5. Besides, the coefficients on L.SGR are positive and significant (0.022, *p*< 0.10; 0.024, *p*< 0.10). Good sales growth stimulates corporate innovation enthusiasm, and enhances innovation efficiency. However, the coefficients on BDS are negative and significant (-0.455, *p*< 0.05; -0.425, *p*< 0.10), which are different from that Balsmeier et al. (2017) [64] believed that independent directors promote corporate innovation. This may be due to the fact that independent directors are hired by the management in China, and there exists the phenomenon of “favor directors.” They often “seek no merit but no fault,” and do not openly challenge the management, which makes it difficult to exert promotion effect on innovation output.

In Model 2, the conclusions on L.R&D, L.LEV, L.ROA, L.TAT, BDS, SHJZ, Age, and STATE are consistent with those from Tables 4 or 5. Besides, the coefficients on L.LnSALARY, and L.AUDIT are positive and significant (0.026, *p*< 0.05; 0.772, *p*< 0.01), suggesting that it is necessary to enhance executives’ diligence and external audit quality, to further mitigate financial mismatch.

## 6. Robustness test

### 6.1 Re-measuring innovation performance

In different industries, the competition degree varies, and the speed of technological upgrading presents heterogeneity, so their innovation capabilities are also different. With reference to Li (2020) [14], an enterprise’s innovation performance is measured by dividing its annual number of patent applications by the average patent applications of the industry in which the enterprise belongs, expressed as M_PATENT, to enhance the comparability of innovation output among enterprises in different industries. Eq (6) presents the calculation method. In the numerator, PATENT is the number of patent applications in the current period. The denominator MPATENT represents the average patent applications of the industry in which the enterprise belongs, and equals to that the number of patent applications of the industry is divided by that of individual enterprises in this industry. Then, M_PATENT is used as the explained variable in Models 1 and 3. In Table 7, columns 1 and 2 present the corresponding regression results.


M_PATENT=PATENTMPATENT (6)

**Table 7:** Regression results for re-measuring innovation performance.

Variable	(1)	(2)	(3)	(4)
	Model 1	Model 3	Model 1	Model 3
	Coef. (S.E.)	Coef. (S.E.)	Coef. (S.E.)	Coef. (S.E.)
Intercept	-9.314*** (2.223)	-9.567*** (2.225)	-13.831*** (0.376)	-13.626*** (0.375)
IC	0.240** (0.102)	0.210** (0.099)	0.676*** (0.114)	0.638*** (0.116)
FMM		-0.098*** (0.023)		-0.116*** (0.024)
L.R&D	0.006 (0.007)	0.006 (0.007)	0.077*** (0.005)	0.075*** (0.005)
L.LEV	-0.002 (0.002)	0.000 (0.002)	0.005*** (0.001)	0.007*** (0.001)
L.ROA	0.005** (0.002)	0.004* (0.002)	0.017*** (0.003)	0.015*** (0.003)
L.TAT	0.241** (0.116)	0.229** (0.115)	0.388*** (0.059)	0.359*** (0.061)
L.SGR	0.006 (0.015)	0.006 (0.014)	0.015 (0.020)	0.018 (0.023)
BDS	-0.913 (0.561)	-0.900 (0.560)	-0.661*** (0.218)	-0.628*** (0.219)
SHJZ	0.124 (0.136)	0.120 (0.135)	-0.215*** (0.056)	-0.239*** (0.055)
Age	0.011 (0.033)	0.013 (0.033)	-0.006** (0.003)	-0.006** (0.003)
L.LnSALARY	0.088 (0.060)	0.089 (0.060)	0.092*** (0.021)	0.095*** (0.020)
L.LnASSET	0.405*** (0.080)	0.414*** (0.080)	0.632*** (0.016)	0.623*** (0.016)
L.AUDIT	0.032 (0.070)	0.024 (0.069)	0.248** (0.106)	0.249** (0.106)
STATE	0.015 (0.077)	0.002 (0.077)	-0.008 (0.031)	-0.034 (0.033)
YEAR/IND	YES	YES	YES	YES
# of obs.	12962	12962	12966	12966
F_Value	8.14***	8.30***		
alpha			(1.429,1.517)	(1.423,1.512)
Wald_chi^2^			5699.43***	5744.70***

Note

*** Significant at 1%

** Significant at 5%

* Significant at 10%.

Robust standard errors in brackets are clustered at corporate level.

Based on the diversification evaluation on innovation output, the number of patent applications (PATENT) is directly adopted to measure innovation performance in Models 1 and 3. PATENT is a non-negative integer, which is a counting variable. And the commonly used counting models involve Poisson regression and Negative binomial regression. In the observations, the standard deviation of PATENT is 312.734, more than 3 times of its mean (79.228). PATENT is excessively discrete, and innovation output is quite differentiated. With reference to Wang and Dai (2019) [5], the Negative binomial regression is taken, to avoid underestimating standard error and overestimating significance. In Table 7, columns 3 and 4 report the results for Models 1 and 3, respectively. Where, the 95% confidence intervals of the excessive dispersion parameter “alpha” are (1.429,1.517) (1.423,1.512), so the hypothesis that the alpha equals to zero corresponding to Poisson regression can be rejected at 5% significance, indicating that the Negative binomial regression is appropriate.

From columns 1 to 4, the coefficients on IC are positive and significant (0.240, *p*< 0.05; 0.210, *p*< 0.05; 0.676, *p*< 0.01; 0.638, *p*< 0.01), showing again that the “reasonable control” hypothesis on IC is suitable for the current situation in China’s companies. In columns 2 and 4, the coefficients on FMM are negative and significant (-0.098, *p*< 0.01; -0.116, *p*< 0.01), implying again that financial mismatch hinders technological innovation.

For the control variables, from columns 1 to 4, the coefficients on L.ROA, L.TAT, and L.LnASSET are statistically significant, and the conclusions on them are consistent with those from Tables 4 or 5. Meanwhile, in columns 3 and 4, the coefficients on L.R&D, L.LEV, BDS, SHJZ, Age, L.LnSALARY, and L.AUDIT are statistically significant, and the conclusions on them are consistent with those from Tables 4 or 6.

## 6.2 Re-measuring IC effectiveness

With stakeholders’ increasing attention, IC standards are more scientific and reasonable. Enterprises’ IC understanding is deepening, and the experience level is gradually improving. And most enterprises’ IC effectiveness tends to improve over time [65]. To alleviate the adverse interference caused by time trend on the results, the DIB · IC index is ranked from low to high based on industry-annual standard, and expressed as IC_R, to re-measure IC effectiveness. The higher the IC_R is, the better the IC effectiveness within same industry and year. On this basis, IC_R is adopted as the explanatory variable in Models 1 to 3, to conduct regression analyses again. Moreover, IC_R is normalized based on industry-annual standard, to reduce the adverse effects of dimensional differences. Table 8 reports the results after re-measuring IC effectiveness.

**Table 8:** Regression results for re-measuring IC effectiveness.

Variable	(1)	(2)	(3)
	Model 1	Model 2	Model 3
	Coef. (S.E.)	Coef. (S.E.)	Coef. (S.E.)
Intercept	12.761*** (0.992)	-2.697*** (0.681)	12.545*** (0.998)
IC_R	0.063** (0.031)	-0.143*** (0.019)	0.052* (0.031)
FMM			-0.080*** (0.021)
L.R&D	0.010 (0.006)	-0.003 (0.002)	0.009 (0.006)
L.LEV	-0.001 (0.001)	0.021*** (0.001)	0.000 (0.001)
L.ROA	0.010*** (0.002)	-0.004*** (0.001)	0.010*** (0.002)
L.TAT	0.007 (0.058)	-0.121*** (0.036)	-0.003 (0.058)
L.SGR	0.004 (0.005)	0.001 (0.009)	0.004 (0.005)
BDS	-0.434 (0.283)	0.135 (0.166)	-0.423 (0.283)
SHJZ	-0.065 (0.053)	-0.039 (0.033)	-0.068 (0.052)
Age	-0.863*** (0.023)	0.021* (0.012)	-0.861*** (0.023)
L.LnSALARY	0.035 (0.022)	0.008 (0.015)	0.036* (0.021)
L.LnASSET	0.391*** (0.039)	0.093*** (0.028)	0.399*** (0.039)
L.AUDIT	0.168** (0.084)	-0.105* (0.061)	0.160* (0.084)
STATE	-0.068 (0.087)	-0.140** (0.058)	-0.079 (0.086)
YEAR/IND	YES	YES	YES
# of obs.	12956	12956	12956
Within_R^2^	0.193	0.186	0.195
F_Value	77.75***	74.23***	76.51***

Note

*** Significant at 1%

** Significant at 5%

* Significant at 10%.

Robust standard errors in brackets are clustered at corporate level.

From Models 1 to 3, the coefficients on IC_R are significant (0.063, *p*< 0.05; -0.143, *p*< 0.01; 0.052, *p*< 0.10). In Model 3, the coefficient on FMM is negative and significant (-0.080, *p*< 0.01). Effective IC mitigates financial mismatch, and promote innovation output. And the mitigation of financial mismatch has a significant mediating effect between effective IC and innovation performance. Again, the results verify Hypotheses 1, 2 and 3 above.

For the control variables, in Models 1 and 3, the conclusions on L.ROA, Age, L.LnSALARY, L.LnASSET, and L.AUDIT are consistent with those from Tables 4 or 5. In Model 2, the conclusions on L.LEV, L.ROA, L.TAT, L.LnASSET, and STATE are consistent with those from Table 4. However, the coefficient on Age is positive and significant (0.021, *p*< 0.10); and that on L.AUDIT is negative and significant (-0.105, *p*< 0.10), which differ from those from Tables 5 and 6. Maybe, financial institutions raise financing costs for recessionary enterprises to prevent disorderly expansion. And positive audit evaluation is helpful to reduce financing costs, and alleviate financial mismatch.

### 6.3 Analyses based on propensity score matching (PSM) sample

Enterprises’ IC effectiveness may be inherent in the environment and own characteristics, so that the characteristic variables in the enterprises with higher IC effectiveness are quite different from those with lower IC effectiveness. If the differences of these characteristic variables affect financial mismatch and innovation performance, then there may be interference factors in the relations among IC, financial mismatch and innovation performance based on full-sample analyses. Therefore, with reference to Li (2022) [49], a dummy variable (MIC) is set as the explained variable in Logit Model 5. If corporate DIB · IC index is greater than its corresponding industry-annual median, this study considers that IC is more effective, and MIC is 1. Otherwise, MIC is set to 0, meaning that IC is less effective. Meanwhile, R&D, LEV, ROA, TAT, SGR, BDS, SHJZ, Age, LnSALARY, LnASSET, AUDIT, STATE, annual and industry dummy variables are selected as covariates to screen out the treatment group and control group. PSM condenses multiple features into a “propensity score” indicator, to facilitate the overall matching of multiple features. Then, pairs are made according to one-to-one nearest neighbor matching, to reduce the influence of possible interference factors. Further, to prompt covariates balance as much as possible, with reference to Chen et al. (2021) [66], this study removes the pairs with absolute difference in p-score exceeding the 75% quantile of absolute differences. Table 9 reports the results for Models 1 to 3 based on PSM sample.

**Table 9:** Regression results based on PSM sample.

Variable	(1)	(2)	(3)
	Model 1	Model 2	Model 3
	Coef. (S.E.)	Coef. (S.E.)	Coef. (S.E.)
Intercept	12.808*** (1.246)	-2.017** (0.891)	12.667*** (1.249)
IC	0.215** (0.097)	-0.441*** (0.062)	0.185* (0.096)
FMM			-0.070*** (0.023)
L.R&D	0.017*** (0.005)	-0.004 (0.003)	0.016*** (0.005)
L.LEV	-0.001 (0.001)	0.025*** (0.001)	0.000 (0.002)
L.ROA	0.012*** (0.003)	-0.005** (0.002)	0.012*** (0.003)
L.TAT	0.037 (0.081)	-0.207*** (0.060)	0.023 (0.081)
L.SGR	0.009 (0.006)	-0.016* (0.008)	0.008 (0.006)
BDS	-0.471 (0.320)	0.149 (0.212)	-0.461 (0.320)
SHJZ	-0.095 (0.096)	-0.041 (0.059)	-0.098 (0.095)
Age	-0.876*** (0.031)	0.034** (0.017)	-0.874*** (0.031)
L.LnSALARY	0.045 (0.028)	-0.012 (0.024)	0.044 (0.028)
L.LnASSET	0.375*** (0.044)	0.068** (0.034)	0.380*** (0.044)
L.AUDIT	0.392* (0.222)	-0.069 (0.144)	0.388* (0.220)
STATE	-0.044 (0.102)	-0.176** (0.071)	-0.056 (0.101)
YEAR/IND	YES	YES	YES
# of obs.	9526	9526	9526
Within_R^2^	0.198	0.201	0.199
F_Value	48.08***	25.52***	45.92***

Note

*** Significant at 1%

** Significant at 5%

* Significant at 10%.

Robust standard errors in brackets are clustered at corporate level.

**Model 5**.


$$\begin{array}{l} MIC_{i,t}=\kappa_{0}+\kappa_{1}R\&D_{i,t-}{}_{1}+\kappa_{2}LEV_{i,t-}{}_{1}+\kappa_{3}ROA_{i,t-}{}_{1}+\kappa_{4}TAT_{i,t-}{}_{1}+\kappa_{5}SGR_{i,t-}{}_{1}+\kappa_{6}BDS_{i,t}+\\ \;\kappa_{7}SHJZ_{i,t}+\kappa_{8}Age_{i,t}+\kappa_{9}LnSALARY_{i,t-}{}_{1}+\kappa_{10}LnASSET_{i,t-}{}_{1}+\kappa_{11}AUDIT_{i,t-}{}_{1}+\\ \;\kappa_{12}STATE_{i,t}+\kappa_{13}\sum_{t}YEAR+\kappa_{14}\sum_{t}IND+\varepsilon_{i,t} \end{array}$$ (7)

From Models 1 to 3, the coefficients on IC are significant (0.215, *p*< 0.05; -0.441, *p*< 0.01; 0.185, *p*< 0.10), again, showing that effective IC enhances innovation performance and alleviates financial mismatch. In Model 3, the coefficient on FMM is negative and significant (-0.070, *p*< 0.01). Financial mismatch means that financial resources are not optimally allocated, which negatively affects innovation output. Based on the above results, once again, this study believes that alleviating financial mismatch is a mediating transmission path that effective IC enhances innovation performance.

For the control variables, in Models 1 and 3, the conclusions on L.R&D, L.ROA, Age, L.LnASSET, and L.AUDIT are consistent with those from Tables 4 or 5. In Model 2, the conclusions on L.LEV, L.ROA, L.TAT, Age, L.LnASSET, and STATE are consistent with those from Tables 4 or 8. However, the coefficient on L.SGR is negative and significant (-0.016, *p*< 0.10), which differs from those from Table 5. Enterprises’ rational growth mitigates operational risks, reduces financing costs, and alleviates financial mismatch.

## 7. Discussion

In China, as an emerging economy, enterprises’ property structure affects operation efficiency. Because of the natural connection for state-owned enterprises with the government, the government usually intervenes in state-owned enterprises’ innovation decisions, to ensure social employment and stabilize economic growth [67]. State-owned enterprises lack competitiveness and motivation in innovation activities. Non-state-owned enterprises have a clear property structure, and face sufficient market competition. The innovation performance of non-state-owned enterprises is higher than that of state-owned enterprises [25], and non-state-owned enterprises are in a leading position in innovation activities. Then, in state-owned and non-state-owned enterprises, does effective IC take a differentiated effect on innovation performance?.

Financial discrimination is common in China’s financial system, and state-owned sectors enjoy preferential allocation of financial resources [68]. Due to the advantages of ownership and scale, state-owned enterprises have convenient access to capital with lower costs, while non-state-owned enterprises suffer from higher capital costs or financing constraints due to their opaque financial information and lack of collateral [68]. Then, does effective IC take differentiated effects on financial mismatch in enterprises with different properties? In the case of financial mismatch, more financial resources flow to state-owned enterprises with stronger innovation inertia, aggravating the financing constraints for innovation activities in non-state-owned enterprises. Then, is the transmission pathway that effective IC mitigates financial mismatch and enhances innovation performance reflected in enterprises with different properties?.

In view of the above problems, this study distinguishes the state-owned (STATE = 1) and non-state-owned (STATE = 0) groups, and conducts fixed effects regression for Model 2. In addition, it is considered that when a company submits a patent application, it can become the real intellectual property owned by the company only after officially authorized. Therefore, based on the consideration of indicator diversity measurement, this study adopts the annual number of patents granted (GPATENT) to measure innovation output in line with Luong et al. (2017) [52], and Li (2020) [14]. On this basis, Models 6 and 7 are constructed, and the properties are differentiated to carry out Negative binomial regression. Since technological innovation is a dynamic and continuous evolution process, there is a time lag in patents authorization, in Models 6 and 7, the values in Period ***t+1*** are taken for the explained variable, and those in Period ***t*** for both explanatory and control variables. In Table 10, columns 1 to 3, and columns 4 to 6 report the results for STATE = 1 and STATE = 0, respectively.

**Table 10:** Results for STATE = 1 and STATE = 0.

Variable	STATE = 1			STATE = 0		
	(1)	(2)	(3)	(4)	(5)	(6)
	Model 2	Model 6	Model 7	Model 2	Model 6	Model 7
	Coef. (S.E.)	Coef. (S.E.)	Coef. (S.E.)	Coef. (S.E.)	Coef. (S.E.)	Coef. (S.E.)
Intercept	0.595 (1.072)	-13.678*** (0.502)	-13.635*** (0.493)	-3.135*** (0.865)	-13.517*** (0.503)	-13.315*** (0.507)
IC	-0.033 (0.066)	0.099 (0.161)	0.100 (0.162)	-0.489*** (0.079)	0.593*** (0.146)	0.528*** (0.147)
FMM			-0.023 (0.039)			-0.117*** (0.020)
L.R&D	-0.002 (0.002)			-0.003 (0.003)		
R&D		0.137*** (0.012)	0.136*** (0.012)		0.047*** (0.005)	0.045*** (0.005)
L.LEV	0.014*** (0.002)			0.024*** (0.001)		
LEV		0.002* (0.001)	0.003** (0.001)		0.005*** (0.001)	0.008*** (0.001)
L.ROA	-0.000 (0.003)			-0.004*** (0.002)		
ROA		0.002 (0.005)	0.002 (0.005)		0.010*** (0.003)	0.008*** (0.003)
L.TAT	-0.095** (0.047)			-0.160*** (0.049)		
TAT		0.501*** (0.066)	0.497*** (0.065)		0.244*** (0.075)	0.216*** (0.071)
L.SGR	-0.014*** (0.003)			0.008 (0.009)		
SGR		-0.010 (0.008)	-0.010 (0.008)		-0.007 (0.012)	-0.006 (0.012)
BDS	0.030 (0.184)	0.015 (0.312)	0.019 (0.312)	0.288 (0.255)	-0.534** (0.272)	-0.447* (0.270)
SHJZ	-0.055 (0.040)	-0.005 (0.085)	-0.011 (0.085)	0.037 (0.070)	-0.131 (0.135)	-0.152 (0.131)
Age	-0.004 (0.016)	-0.006 (0.004)	-0.006 (0.004)	0.030* (0.018)	-0.006** (0.003)	-0.005* (0.003)
L.LnSALARY	0.004 (0.017)			0.001 (0.024)		
LnSALARY		0.072*** (0.027)	0.072*** (0.027)		0.166*** (0.022)	0.159*** (0.022)
L.LnASSET	-0.038 (0.044)			0.129*** (0.036)		
LnASSET		0.681*** (0.020)	0.679*** (0.019)		0.588*** (0.022)	0.584*** (0.022)
L.AUDIT	-0.030 (0.098)			-0.090 (0.071)		
AUDIT		0.360** (0.156)	0.363** (0.156)		0.161 (0.120)	0.177 (0.119)
YEAR/IND	YES	YES	YES	YES	YES	YES
# of obs.	4666	4631	4631	8315	8350	8350
*R* ^2^	0.115			0.230		
alpha		(1.319,1.450)	(1.318,1.450)		(1.348,1.447)	(1.341,1.439)
F_value	16.66***			64.26***		
Wald_chi^2^		3659.69***	3784.14***		3987.23***	3994.25***

Note

*** Significant at 1%

** Significant at 5%

* Significant at 10%.

Robust standard errors in brackets are clustered at corporate level.

**Model 6**.


$$\begin{array}{l} GPATENT_{i,t+1}=\lambda_{0}+\lambda_{1}IC_{i,t}+\lambda_{2}R\&D_{i,t}+\lambda_{3}LEV_{i,t}+\lambda_{4}ROA_{i,t}+\lambda_{5}TAT_{i,t}+\lambda_{6}SGR_{i,t}+\lambda_{7}BDS_{i,t}+\\ \;\lambda_{8}SHJZ_{i,t}+\lambda_{9}Age_{i,t}+\lambda_{10}LnSALARY_{i,t}+\lambda_{11}LnASSET_{i,t}+\lambda_{12}AUDIT_{i,t}+\\ \;\lambda_{13}\sum_{t}YEAR+\lambda_{14}\sum_{t}IND+\varepsilon_{i,t} \end{array}$$ (8)

**Model 7**.


$$\begin{array}{l} GPATENT_{i,t+1}=\theta_{0}+\theta_{1}IC_{i,t}+\theta_{2}FMM_{i,t}+\theta_{3}R\&D_{i,t}+\theta_{4}LEV_{i,t}+\theta_{5}ROA_{i,t}+\theta_{6}TAT_{i,t}+\theta_{7}SGR_{i,t}+\\ \;\theta_{8}BDS_{i,t}+\theta_{9}SHJZ_{i,t}+\theta_{10}Age_{i,t}+\theta_{11}LnSALARY_{i,t}+\theta_{12}LnASSET_{i,t}+\\ \;\theta_{13}AUDIT_{i,t}+\theta_{14}\sum_{t}YEAR+\theta_{15}\sum_{t}IND+\varepsilon_{i,t} \end{array}$$ (9)

For Model 2, in column 1, the coefficient on IC is not statistically significant (-0.033, *p>* 0.10); in column 4, that is negative and significant (-0.489, *p*< 0.01). The mitigation effect of effective IC on financial mismatch is reflected in non-state-owned enterprises, but not in state-owned enterprises. In China, the “dual” ownership allocation of financial resources is obvious, and a large number of financial resources are tilted towards state-owned enterprises [68,69]. Due to the financing advantage from the natural connection with the government, state-owned enterprises are usually not burdened by financial mismatch. However, non-state-owned enterprises are often faced with financing disadvantage, and are the main undertakers for financial mismatch. Compared with state-owned enterprises, in non-state-owned enterprises, effective IC has a significant marginal mitigating effect on financial mismatch. Heterogeneity in credit conditions is important in accounting for efficiency gains [70]. Therefore, the regulators should create a reasonable financial ecological environment, to reduce ownership discrimination in resources allocation.

For Models 6 and 7, with STATE = 1, the coefficients on IC are not statistically significant (0.099, *p>* 0.10; 0.100, *p>* 0.10); with STATE = 0, those are positive and significant (0.593, *p*< 0.01; 0.528, *p*< 0.01). The leaders within state-owned enterprises are often appointed by the government, and effective checks and balances cannot be carried out between the governance and the management. As a result, IC cannot play a positive role fully, which is not conducive to improving innovation activities, and it is difficult to take a positive effect on innovation output. Non-state-owned enterprises take the initiative to improve IC effectiveness based on the market environment and corporate development [71]. Further, the positive effect of effective IC can be fully brought into play to enhance innovation performance. Compared with state-owned enterprises, in non-state-owned enterprises, effective IC contributes to a significant increase in innovation output [14]. For Model 7, with STATE = 1, the coefficient on FMM is not statistically significant (-0.023, *p>* 0.10); with STATE = 0, that is negative and significant (-0.117, *p*< 0.01). Financial mismatch may cause a resource “crowding out effect” on non-state-owned enterprises with higher innovation efficiency. Non-state-owned enterprises are troubled by “difficult and expensive financing” [72], leading to insufficient R&D input, hindering technological R&D activities, and inhibiting innovation output.

Based on Models 2, 6 and 7, the mechanism that effective IC mitigates financial mismatch, and enhances innovation output is reflected significantly in non-state-owned enterprises, but not in state-owned enterprises. State-owned enterprises enjoy external credit financing advantage [68,69], and the soft budget constraints and weak supervision lead to the lack of the managers’ motivation to pursue efficiency [73]. In contrast, non-state-owned enterprises have a relatively sound supervision mechanism, which is easier to enhance IC effectiveness, and improve innovation efficiency while alleviating financial mismatch.

For the control variables, in Model 2, the conclusions on L.LEV, L.ROA, L.TAT, L.SGR, Age, and L.LnASSET are consistent with those from Tables 4, 8 or 9. In Models 6 and 7, the conclusions on R&D, LEV, ROA, TAT, BDS, Age, LnSALARY, LnASSET, and AUDIT are consistent with those from Table 4, 5 or 6 above.

## 8. Conclusion and recommendation

### 8.1 Conclusions

From the perspective of IC and financial mismatch jointly affecting technological innovation, based on the data of listed companies in China from 2012 to 2020, this study explains the effects of IC on financial resource allocation and corporate innovation, to deepen the understanding of IC and financial mismatch affecting technological innovation. The results show that effective IC plays a significant role in enhancing innovation performance, supporting the “reasonable control” hypothesis that effective IC promotes corporate innovation; effective IC alleviates “difficult and expensive financing”, fully mitigates the financial mismatch faced by enterprises; the mitigation of financial mismatch presents a significant mediating effect between effective IC and innovation performance. Mitigating financial mismatch is an important pathway that effective IC improves technological innovation. Further, the endogeneity is weakened by Heckman two-step model and instrumental variable method; and robustness tests are performed by re-measuring innovation performance, IC effectiveness, and based on PSM sample. Again, the above conclusions are verified. In Discussion, the results show that effective IC facilitates innovation performance, and mitigates financial mismatch in non-state-owned enterprises, but not in state-owned enterprises. And the transmission mechanism that effective IC alleviates financial mismatch, and enhances innovation out is reflected in non-state-owned enterprises, but not in state-owned enterprises.

Based on the impacts of IC on innovation output and financial mismatch, this study explores the transmission mechanism of financial mismatch mitigation for the positive effect of effective IC on innovation performance, enriching the research on IC economic consequences and influencing factors for innovation performance. This study has important theoretical and practical significance. In theoretical side, this study explains the impact of effective IC on innovation performance, and explores the mediating pathway that effective IC alleviates financial mismatch and enhances innovation out, thereby enriches the literatures on the mechanism that IC influences innovation efficiency. On practical side, this study provides empirical evidence for the regulators to reinforce supervision and inspection, to boost IC construction, and better facilitate corporate innovation. Meanwhile, this study provides decision support for enterprises with different properties to improve IC effectiveness, mitigate financial mismatch and enhance innovation efficiency, to promote the development of the real economy.

### 8.2 Recommendations

At present, China is in the critical period from high-speed growth to high-quality development [9]. Enterprises take measures such as improving internal supervision mechanism, and optimizing internal governance structure to ensure IC quality, and create unique competitive advantages. IC mechanism is constantly improved, and the important role of effective IC in innovation is brought into play, to stimulate corporate innovation potential, enhance innovation output, and promote the achievement of strategic objectives. IC effectiveness is continuously improved, and effective IC is implemented in daily operation activities. IC governance role should be brought into play to improve credit system and information disclosure, to alleviate external financing constraints, make interest rates truly reflect the supply and demand in the financial factor market, broaden financing channels, optimize resource allocation, and facilitate financial resources towards the enterprises with good development prospects.

The allocation of financial resources should be further improved, to promote the efficiency of financial services to the real economy. The easing of capital misallocation and enhancement of financial sector’s ability to serve the real economy are intrinsic requirements for high-quality economic development. Financial system should be improved to give full play to the key role of financial market in resource allocation. The allocation of financial resources is continuously optimized, and the threshold of financial services should be lowered, to give better play to the role in promoting technological innovation and fostering economic momentum. The regulatory authorities promote enterprises to improve IC standards, reinforce IC information disclosure, and facilitate the role of effective IC in alleviating financial mismatch and enhancing innovation performance, to form a virtuous cycle supporting innovation.

The regulators provide innovation guarantee services for enterprises with different properties. The capital, insurance and currency markets should be further opened up, and diversified financing support should be provided for technological innovation. The reform of state-owned enterprises should be deepened continuously, to avoid empty ownership, further improve the modern property system, and give play to the positive impact of IC on technological innovation. The government reduces hidden subsidies to state-owned enterprises, and forces them to carry out innovation activities through market competition, to stimulate innovation potential and get rid of innovation inertia. In state-owned enterprises, the long-term driving mechanism of IC to reduce financial mismatch and promote technological innovation should be formed and accelerated. Non-state-owned enterprises should be given sufficient development space to achieve the optimal allocation of financial resources, so as to better boost their innovation activities. The quality of technological innovation should be enhanced, and the innovation activities in state-owned and non-state-owned enterprises should be coordinated to promote the steady and healthy development of the economy.

### 8.3 Limitations and prospects

Innovation is both an output and a process. It is a production, or adoption and assimilation that can generate value, including product and service update and market expansion, as well as the development of new production methods, and the realization of new management systems [74]. Limited to data availability, this study only adopts patents output to measure innovation performance, which may not be a comprehensive evaluation on corporate innovation. In future research, more data sources can be considered, such as the output or sales volume of new products, market share of products or services, etc., to comprehensively measure innovation performance, so as to obtain more extensive empirical evidence. Besides, due to space constraints, this study only explores the transmission mechanism among effective IC, financial mismatch and innovation performance. In the future, the joint effect mechanism of external governance factors such as institutional shareholding, media attention, and IC as an internal governance factor on innovation activities can be considered, in order to accelerate the pace of building an innovation-oriented economy, which may be a major research prospect.

## Supporting information

S1 Dataset The data set used in this article for discussion and analysis. (ZIP)
