# Correction: Corporate internal control, financial mismatch mitigation and innovation performance

**DOI:** 10.1371/journal.pone.0316123

**Published:** 2024-12-16

**Authors:** Xiao Li, Zhiquan Zhao

There are errors in the Funding statement. The correct Funding statement is as follows: This work is supported in part by the National Social Science Fund of China (20BGL026 and 18BGL237), the Soft Science Research Program of Henan Province, China (222400410161), and the Key Research Institute of Humanities and Social Sciences at Universities of Henan, China.
