# Supplementary material for: Corporate internal control, financial mismatch mitigation and innovation performance
Source: PLoS One. 2022 Dec 27;17(12):e0278633. doi: 10.1371/journal.pone.0278633 (PMC9794094; doi:10.1371/journal.pone.0278633)
Supplement: S1 Dataset — (ZIP) [file pone.0278633.s001.zip › S1 Dataset/Alleviating Endogeneity 1/Alleviating endogeneity 1.docx]

**Alleviating Endogeneity 1**

**Heckman Two-Step Model**

bysort code: gen IC4=IC[_n-1]

gen DIC=(IC>IC4)

**Model 1.**

heckman LnINVENT ICA L.RD L.LEV L.ROA L.TAT L.SGR BDS SHJZ Age L.LnSALARY L.LnASSET L.AUDIT STATE dum_yr* dum_ind*, select(DIC= L.RD L.LEV L.ROA L.TAT L.SGR BDS SHJZ Age L.LnSALARY L.LnASSET L.AUDIT STATE dum_yr* dum_ind* L.Big4 L.Isviolated) twostep nolog

**Model 2.**

heckman FMM ICA L.RD L.LEV L.ROA L.TAT L.SGR BDS SHJZ Age L.LnSALARY L.LnASSET L.AUDIT STATE dum_yr* dum_ind*, select(DIC= L.RD L.LEV L.ROA L.TAT L.SGR BDS SHJZ Age L.LnSALARY L.LnASSET L.AUDIT STATE dum_yr* dum_ind* L.Big4 L.Isviolated) twostep nolog

**Model 3.**

heckman LnINVENT ICA FMM L.RD L.LEV L.ROA L.TAT L.SGR BDS SHJZ Age L.LnSALARY L.LnASSET L.AUDIT STATE dum_yr* dum_ind*, select(DIC= L.RD L.LEV L.ROA L.TAT L.SGR BDS SHJZ Age L.LnSALARY L.LnASSET L.AUDIT STATE dum_yr* dum_ind* L.Big4 L.Isviolated) twostep nolog
