# Supplementary material for: Corporate internal control, financial mismatch mitigation and innovation performance
Source: PLoS One. 2022 Dec 27;17(12):e0278633. doi: 10.1371/journal.pone.0278633 (PMC9794094; doi:10.1371/journal.pone.0278633)
Supplement: S1 Dataset — (ZIP) [file pone.0278633.s001.zip › S1 Dataset/Alleviating Endogeneity 2/Alleviating endogeneity 2.docx]

**Alleviating Endogeneity 2**

**Instrumental Variable Method**

**Model 1.**

ivregress 2sls LnPATENT L.RD L.LEV L.ROA L.TAT L.SGR BDS SHJZ Age L.LnSALARY L.LnASSET L.AUDIT STATE dum_yr* dum_ind* (ICA = L.Big4 L.Isviolated), first r

**Model 2.**

ivregress 2sls FMM L.RD L.LEV L.ROA L.TAT L.SGR BDS SHJZ Age L.LnSALARY L.LnASSET L.AUDIT STATE dum_yr* dum_ind* (ICA = L.Big4 L.Isviolated), first r

**Model 3.**

ivregress 2sls LnPATENT FMM L.RD L.LEV L.ROA L.TAT L.SGR BDS SHJZ Age L.LnSALARY L.LnASSET L.AUDIT STATE dum_yr* dum_ind* (ICA = L.Big4 L.Isviolated), first r
