# Supplementary material for: Corporate internal control, financial mismatch mitigation and innovation performance
Source: PLoS One. 2022 Dec 27;17(12):e0278633. doi: 10.1371/journal.pone.0278633 (PMC9794094; doi:10.1371/journal.pone.0278633)
Supplement: S1 Dataset — (ZIP) [file pone.0278633.s001.zip › S1 Dataset/Descriptive Statistics and Correlation/Descriptive Statistics and Correlation.docx]

**1. Descriptive statistics**

tabstat LnPATENT ICA FMM RD LEV ROA TAT SGR BDS SHJZ Age LnSALARY LnASSET AUDIT STATE, stat(mean median max min sd count) col(stat)

**2. Correlation**

pwcorr LnPATENT ICA FMM RD LEV ROA TAT SGR BDS SHJZ Age LnSALARY LnASSET AUDIT STATE, sig star(0.1)
