# Supplementary material for: Corporate internal control, financial mismatch mitigation and innovation performance
Source: PLoS One. 2022 Dec 27;17(12):e0278633. doi: 10.1371/journal.pone.0278633 (PMC9794094; doi:10.1371/journal.pone.0278633)
Supplement: S1 Dataset — (ZIP) [file pone.0278633.s001.zip › S1 Dataset/Discussion/Discussion.docx]

**Discussion**

**1. if STATE==1**

**Model 2.**

xtreg FMM ICA L.RD L.LEV L.ROA L.TAT L.SGR BDS SHJZ Age L.LnSALARY L.LnASSET L.AUDIT dum_yr* dum_ind* if STATE==1, fe r

**Model 6.**

nbreg F.PATENT ICA RD LEV ROA TAT SGR BDS SHJZ Age LnSALARY LnASSET AUDIT dum_yr* dum_ind* if STATE==1, r

**Model 7.**

nbreg F.PATENT ICA FMM RD LEV ROA TAT SGR BDS SHJZ Age LnSALARY LnASSET AUDIT dum_yr* dum_ind* if STATE==1, r

**2. if STATE==0**

**Model 2.**

xtreg FM ICA L.RD L.LEV L.ROA L.TAT L.SGR BDS SHJZ Age L.LnSALARY L.LnASSET L.AUDIT dum_yr* dum_ind* if STATE==0, fe r

**Model 6.**

nbreg F.PATENT ICA RD LEV ROA TAT SGR BDS SHJZ Age LnSALARY LnASSET AUDIT dum_yr* dum_ind* if STATE==0, r

**Model 7.**

nbreg F.PATENT ICA FMM RD LEV ROA TAT SGR BDS SHJZ Age LnSALARY LnASSET AUDIT dum_yr* dum_ind* if STATE==0, r
