# Supplementary material for: Corporate internal control, financial mismatch mitigation and innovation performance
Source: PLoS One. 2022 Dec 27;17(12):e0278633. doi: 10.1371/journal.pone.0278633 (PMC9794094; doi:10.1371/journal.pone.0278633)
Supplement: S1 Dataset — (ZIP) [file pone.0278633.s001.zip › S1 Dataset/Main Regression/Main Regression.docx]

**1. Main Regression**

**Model 1.**

xtreg LnPATENT ICA L.RD L.LEV L.ROA L.TAT L.SGR BDS SHJZ Age L.LnSALARY L.LnASSET L.AUDIT STATE dum_yr* dum_ind*, fe r

**Model 2.**

xtreg FMM ICA L.RD L.LEV L.ROA L.TAT L.SGR BDS SHJZ Age L.LnSALARY L.LnASSET L.AUDIT STATE dum_yr* dum_ind*, fe r

**Model 3.**

xtreg LnPATENT ICA FMM L.RD L.LEV L.ROA L.TAT L.SGR BDS SHJZ Age L.LnSALARY L.LnASSET L.AUDIT STATE dum_yr* dum_ind*, fe r

**2. Confidence interval**

bootstrap r(ind_eff) r(dir_eff), reps(5000): sgmediation LnPATENT,mv(FMM) iv(ICA) cv(RD1 LEV1 ROA1 TAT1 SGR1 BDS SHJZ Age LnSALARY1 LnASSET1 AUDIT1 STATE dum_yr* dum_ind*)

estat bootstrap, percentile bc
