# Supplementary material for: Corporate internal control, financial mismatch mitigation and innovation performance
Source: PLoS One. 2022 Dec 27;17(12):e0278633. doi: 10.1371/journal.pone.0278633 (PMC9794094; doi:10.1371/journal.pone.0278633)
Supplement: S1 Dataset — (ZIP) [file pone.0278633.s001.zip › S1 Dataset/Robustness Test 1/Robustness Test 1.docx]

**Re-measuring Corporate Innovation Performance**

**1. Adopting the relative number of patent applications**

bysort ind year: egen MPATENT=mean(PATENT)

gen M_PATENT= PATENT/MPATENT

xtset code1 year

**Model 1.**

xtreg M_PATENT ICA L.RD L.LEV L.ROA L.TAT L.SGR BDS SHJZ Age L.LnSALARY L.LnASSET L.AUDIT STATE dum_yr* dum_ind*, fe r

**Model 3.**

xtreg M_PATENT ICA FMM L.RD L.LEV L.ROA L.TAT L.SGR BDS SHJZ Age L.LnSALARY L.LnASSET L.AUDIT STATE dum_yr* dum_ind*, fe r

**2. Directly adopting the number of patent applications**

**Model 1.**

nbreg PATENT ICA L.RD L.LEV L.ROA L.TAT L.SGR BDS SHJZ Age L.LnSALARY L.LnASSET L.AUDIT STATE dum_yr* dum_ind*, r

**Model 3.**

nbreg PATENT ICA FMM L.RD L.LEV L.ROA L.TAT L.SGR BDS SHJZ Age L.LnSALARY L.LnASSET L.AUDIT STATE dum_yr* dum_ind*, r
