# Supplementary material for: Corporate internal control, financial mismatch mitigation and innovation performance
Source: PLoS One. 2022 Dec 27;17(12):e0278633. doi: 10.1371/journal.pone.0278633 (PMC9794094; doi:10.1371/journal.pone.0278633)
Supplement: S1 Dataset — (ZIP) [file pone.0278633.s001.zip › S1 Dataset/Robustness Test 2/Robustness Test 2.docx]

**Re-measuring IC effectiveness**

bysort ind year: egen IC_R= rank(IC)

**IC_R normalization**

bysort ind year: egen MAXIC_R=max(IC_R)

bysort ind year: egen MINIC_R=min(IC_R)

gen IC_RA=(IC_R - MINIC_R)/ (MAXIC_R - MINIC_R)

xtset code1 year

**Model 1.**

xtreg LnPATENT IC_RA L.RD L.LEV L.ROA L.TAT L.SGR BDS SHJZ Age L.LnSALARY L.LnASSET L.AUDIT STATE dum_yr* dum_ind*, fe r

**Model 2.**

xtreg FMM IC_RA L.RD L.LEV L.ROA L.TAT L.SGR BDS SHJZ Age L.LnSALARY L.LnASSET L.AUDIT STATE dum_yr* dum_ind*, fe r

**Model 3.**

xtreg LnPATENT IC_RA FMM L.RD L.LEV L.ROA L.TAT L.SGR BDS SHJZ Age L.LnSALARY L.LnASSET L.AUDIT STATE dum_yr* dum_ind*, fe r
