# Supplementary material for: Corporate internal control, financial mismatch mitigation and innovation performance
Source: PLoS One. 2022 Dec 27;17(12):e0278633. doi: 10.1371/journal.pone.0278633 (PMC9794094; doi:10.1371/journal.pone.0278633)
Supplement: S1 Dataset — (ZIP) [file pone.0278633.s001.zip › S1 Dataset/Robustness Test 3/Robustness Test 3.docx]

**Robustness Test 3.**

tabstat _pdif, stat(p75) col(stat)

bysort ind year: egen IC50= pctile(IC), p(50)

gen MIC=(IC>IC50)

xtset code1 year

gen t= MIC

set seed 10101

gen ranorder = runiform( )

sort ranorder

**Model 1.**

psmatch2 t RD1 LEV1 ROA1 TAT1 SGR1 BDS SHJZ Age LnSALARY1 LnASSET1 AUDIT1 STATE dum_yr* dum_ind*, outcome(LnPATENT) n(1) ate ties logit common noreplacement

xtset code1 year

xtreg LnPATENT ICA L.RD L.LEV L.ROA L.TAT L.SGR BDS SHJZ Age L.LnSALARY L.LnASSET L.AUDIT STATE dum_yr* dum_ind* if _weight==1&_pdif<0.065, fe r

**Model 2.**

psmatch2 t RD1 LEV1 ROA1 TAT1 SGR1 BDS SHJZ Age LnSALARY1 LnASSET1 AUDIT1 STATE dum_yr* dum_ind*, outcome(FMM) n(1) ate ties logit common noreplacement

xtset code1 year

xtreg FMM ICA L.RD L.LEV L.ROA L.TAT L.SGR BDS SHJZ Age L.LnSALARY L.LnASSET L.AUDIT STATE dum_yr* dum_ind* if _weight==1&_pdif<0.065, fe r

**Model 3.**

psmatch2 t RD1 LEV1 ROA1 TAT1 SGR1 BDS SHJZ Age LnSALARY1 LnASSET1 AUDIT1 STATE dum_yr* dum_ind*, outcome(LnPATENT) n(1) ate ties logit common noreplacement

xtset code1 year

xtreg LnPATENT ICA FMM L.RD L.LEV L.ROA L.TAT L.SGR BDS SHJZ Age L.LnSALARY L.LnASSET L.AUDIT STATE dum_yr* dum_ind* if _weight==1&_pdif<0.065, fe r
